# Spatio-temporal biodistribution of ^89^Zr-oxine labeled huLym-1-A-BB3z-CAR T-cells by PET imaging in a preclinical tumor model

**DOI:** 10.1038/s41598-021-94490-0

**Published:** 2021-07-23

**Authors:** Naomi S. Sta Maria, Leslie A. Khawli, Vyshnavi Pachipulusu, Sharon W. Lin, Long Zheng, Daniel Cohrs, Xiaodan Liu, Peisheng Hu, Alan L. Epstein, Russell E. Jacobs

**Affiliations:** 1 Department of Physiology and Neuroscience, Keck School of Medicine, University of Southern California, 1501 San Pablo Street, ZNI 117, Los Angeles, CA 90033 USA; 2 Department of Pathology, Keck School of Medicine, University of Southern California, 2011 Zonal Ave, HMR 205, Los Angeles, CA 9033 USA

**Keywords:** Cancer immunotherapy, Targeted therapies

## Abstract

Quantitative in vivo monitoring of cell biodistribution offers assessment of treatment efficacy in real-time and can provide guidance for further optimization of chimeric antigen receptor (CAR) modified cell therapy. We evaluated the utility of a non-invasive, serial ^89^Zr-oxine PET imaging to assess optimal dosing for huLym-1-A-BB3z-CAR T-cell directed to Lym-1-positive Raji lymphoma xenograft in NOD Scid-IL2Rgamma^null^ (NSG) mice. In vitro experiments showed no detrimental effects in cell health and function following ^89^Zr-oxine labeling. In vivo experiments employed simultaneous PET/MRI of Raji-bearing NSG mice on day 0 (3 h), 1, 2, and 5 after intravenous administration of low (1.87 ± 0.04 × 10^6^ cells), middle (7.14 ± 0.45 × 10^6^ cells), or high (16.83 ± 0.41 × 10^6^ cells) cell dose. Biodistribution (%ID/g) in regions of interests defined over T1-weighted MRI, such as blood, bone, brain, liver, lungs, spleen, and tumor, were analyzed from PET images. Escalating doses of CAR T-cells resulted in dose-dependent %ID/g biodistributions in all regions. Middle and High dose groups showed significantly higher tumor %ID/g compared to Low dose group on day 2. Tumor-to-blood ratios showed the enhanced extravascular tumor uptake by day 2 in the Low dose group, while the Middle dose showed significant tumor accumulation starting on day 1 up to day 5. From these data obtained over time, it is apparent that intravenously administered CAR T-cells become trapped in the lung for 3–5 h and then migrate to the liver and spleen for up to 2–3 days. This surprising biodistribution data may be responsible for the inactivation of these cells before targeting solid tumors. Ex vivo biodistributions confirmed in vivo PET-derived biodistributions. According to these studies, we conclude that in vivo serial PET imaging with ^89^Zr-oxine labeled CAR T-cells provides real-time monitoring of biodistributions crucial for interpreting efficacy and guiding treatment in patient care.

## Introduction

Chimeric antigen receptor (CAR) T-cells express genetically modified receptors or antibodies that specifically recognize and bind tumor antigens and trigger a tumor cell killing cascade^1^. Recent CAR developments revolutionized the treatment of hematologic malignancies, leading to current US FDA approved CD19-directed CAR T-cell immunotherapy (*e.g.* Kymirah and Yescarta)^2^. Long term persistence and function of CARs are extremely important for their success in the clinic. Thus, non-invasive evaluation of CAR biodistribution can be important in the development of next generation cell therapies. Positron emission tomography (PET) and magnetic resonance imaging (MRI) can provide this necessary non-invasive and real-time assessment of adoptively transferred cell biodistribution and trafficking. PET/MRI is readily translated to the clinic during therapy to assess the patient’s real-time treatment response and help plan optimal adjustments, including the site of administration and dosing. PET imaging, specifically, provides highly sensitive and quantitative information about adoptive cell biodistribution over the short and long-term using radionuclide probes, and MRI offers high resolution of soft tissues for better defined cell spatial biodistribution.

Direct ex vivo cell labeling has been previously reported with PET radiolabeled complexes (^18^F-FDG, ^64^Cu, ^111^Indium, and ^89^Zr). The long half-lives of ^64^Cu (12.7 h, β +  = 17.9%) and ^89^Zr (78.41 h, β +  = 22.3%) makes them highly suitable for monitoring cell trafficking and tumor response up to 7 days after adoptive transfer. Copper-64 can be bound to drugs, cells or antibodies^3–6^. Zirconium-89 (^89^Zr) chelated with 8-hydroxiquinoline (oxine) or p-isothiocyanatobenzyl-desferrioxamine (DFO-Bz-NCS, Desferal, DFO) has been used to label different types of cells including human CAR T-cells^7,8^, human gamma-delta T-cells, murine cytolytic T lymphocytes^9–11^, murine bone marrow cells^11–13^, murine dendritic and NK cells^11^, rhesus monkeys NK cells^14^, mesenchymal stromal cells^15^ and bound to proteins expressed on immune cell surfaces^16^.

We chose ^89^Zr as the radiolabel due to its longer half-life and oxine as the chelator complex and ionophore. The favored oxidation state of zirconium is 4+ and tetravalent zirconium forms ZrL_4_ complexes with monobasic bidentate ligands such as oxinate, tropolonate, and hydroxamates, where L is defined as the ligand of interest. For ^89^Zr-oxine, ^89^Zr is complexed by four oxinate molecules. The ^89^Zr metal center is eight coordinated in approximately dodecahedral geometry by four chelating N,O pairs from four bidentate oxinate ligands and has been shown to be neutral lipophilic^17,18^. Measurements of ^89^Zr-oxine cell specific activity show that the complex remains intracellularly. This process is analogous to indium, which favors an oxidation state of 3+, forming InL_3_ complexes and is currently clinically available as ^111^InL_3_. Additionally, a one-step kit formulation to prepare ^89^Zr-oxine has been developed and showed that intracellular retention and cell viability of ^89^Zr-oxine is similar to ^111^In-oxine in whole blood cells^19^, facilitating clinical translation of cellular labeling using ^89^Zr-oxine.

While previous studies demonstrated feasibility of ^89^Zr labeling of their cells of interest and accumulation at their target, these studies also showed off-target accumulation of the radiolabeled cells after adoptive transfer in vivo. In this experiment, we investigated whether, over time, the amount of adoptively transferred CAR T-cells correlated with accumulation at the tumor target and at off-target sites, such as liver, spleen, bone and brain, and therefore, provide information about the efficacy of the immunotherapy dose. Three dose levels were chosen (approximately 1 million, 5 million, and ≥ 10 million cells) to address whether administering higher doses would result in more cells accumulating at the target sooner or not because more cells are trapped in off-target sites, or whether a lower dose (~ 1 million cells) would demonstrate efficacy due to less cells getting impinged at off-target regions and, thus, more cells could readily accumulate at the tumor target. For translational aims, we strive to minimize dose and correlate biodistribution with efficacy, and to find optimal temporal targeting of the therapy.

Here, NSG mice were inoculated with non-Hodgkin Raji lymphoma cells subcutaneously and were then intravenously (IV) administered different doses of ^89^Zr-oxine labeled huLym-1-A-BB3z-CAR T-cells. HuLym-1-A is a humanized version of chimeric murine Lym-1 antibody that has been shown to bind to a discontinuous epitope (Lym-1) on several subtypes of HLA-DR with lower affinity in Raji and other forms of non-Hodgkin B cell lymphoma. Furthermore, Lym-1 does not cross-react with mouse MHC-II antigen, which prevents it from affecting or influencing tissue biodistribution in the mouse, thus giving us clear results^20^. In our previous studies, we used a metastatic Raji model in which lymphoma cells were administered intravenously and found that the CAR T-cells potently cleared tumor from all sites, including the brain and the lungs, in the NSG mice^21,22^. In this study, we chose a subcutaneous model to visualize and quantitate tumor uptake of CAR T-cells at a pre-defined region in the mouse distant from other organs and correlate uptake with the CAR T-cell dose. In vivo biodistribution of the ^89^Zr-oxine labeled CAR T-cells were acquired using simultaneous PET/MRI up to 5 days following administration. Invasive ex vivo biodistribution was also performed as an endpoint to complement the in vivo PET imaging study arm.

## Methods

### Ethical approval

The experiment was performed in accordance with the US National Institute of Health guidelines. All procedures were approved by the Institutional Animal Care and Use Committee (IACUC protocols 20658 and 20697) at the University of Southern California. The study was carried out in compliance with the ARRIVE guidelines (https://arriveguidelines.org/).

### Animals, housing, and husbandry

Twelve 6–8 week-old male NOD Scid-IL2Rgamma^null^ (NSG) mice, weighing 23–29 g, used in the study were bred in the USC animal facility (IACUC protocol 20697). Animals were housed with standardized 12 h light and dark cycles and had access to food and water ad libitum. Vivarium temperature was maintained between 22 and 24 °C and humidity between 50 and 60%. The immunodeficiency of NSG mice allowed for the engraftment of the human Burkitt’s lymphoma cell line to provide an in vivo tumor model.

### Raji xenograft in NSG mice

The Raji cell line (Raji-eGFP/Luc) was a gift from Dr Yvonne Y. Chen at the UCLA, and was grown in RPMI 1640 (Irvine Scientific, Irvine, CA) supplemented with 10% fetal bovine serum (Hyclone Laboratories, Logan, UT), L-glutamine, penicillin G (100 units/mL), and streptomycin (100 ug/mL). NSG mice were injected with a 0.1 mL inoculum consisting of 4 × l0^6^ Raji cells in sterile phosphate buffered saline (PBS) subcutaneously in the right flank. The tumors were grown for 12 days prior to ^89^Zr-oxine labeled CAR T-cell injection, resulting in tumor volumes of 100–150 mm^3^. NSG mice with Raji tumor volumes < 50 mm3 measured by caliper palpation were excluded on CAR T-cell injection day. One mouse had a tumor volume of < 50 mm^3^ and was excluded in the study and did not receive a CAR T-cell dose.

### Study design

**Figure 1:**
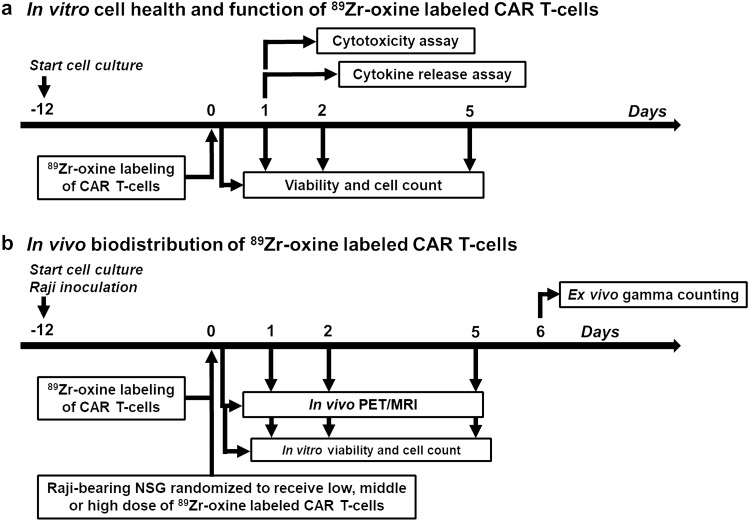
Study timelines. (**a**) In vitro measurements of cell health and function of ^89^Zr labeled and unlabeled non-transduced (mock) T-cells and CAR T-cells were obtained in a separate experimental trial. Percent lysis of target Raji cells (cytotoxicity) and released cytokine levels in situ by the effector ^89^Zr-oxine labeled and unlabeled mock and CAR T-cells were measured 24 h after incubation of the effector cells with the target cells. Incubation of effector and target cells began 3 h post ^89^Zr-oxine labeling. Aliquots of ^89^Zr-oxine labeled and unlabeled mock and CAR T-cells were subcultured for in vitro viability and live cell number counts on the same in vivo scan timepoints. (**b**) In vivo biodistribution measurement of ^89^Zr-oxine labeled CAR T-cells began with CAR T-cell preparation and inoculation of NSG mice with Raji cells 12 days prior ^89^Zr-oxine labeling and IV administration. NSG mice were randomized to receive either a low (126.7 ± 2.5 kBq, 1.87 ± 0.04 × 10^6^ cells, n = 3 mice), middle (542.6 ± 92.1 kBq, 7.14 ± 0.45 × 10^6^ cells, n = 5 mice), or high (1491.7 ± 36.1 kBq, 16.83 ± 0.41 × 10^6^ cells, n = 3 mice) dose of ^89^Zr-oxine labeled huLym-1-BB3z-CAR T-cells and underwent PET/MRI scans on day 0, 1, 2, and 5 time points, which corresponded to 3, 24, 48, and 120 h after CAR T-cell injection. Activity concentrations were obtained from PET scans and expressed as percent injected dose per gram of tissue of manually defined regions of interests that were identified on T1-weighted MRI anatomical scans. Coplanar PET and MRI scans were acquired simultaneously and were registered to the same space. Invasive ex vivo biodistribution was performed on day 6 post injection to complement the in vivo PET imaging study arm. ^89^Zr-oxine labeled and unlabeled CAR T-cells were subcultured for in vitro viability and live cell number counts on the same scan timepoints.

The study design for the in vitro assessment of CAR T-cell health and function following ^89^Zr-oxine labeling is shown in Fig. 1a and was performed in a separate experimental trial. Primary human T-cells were prepared and transduced beginning 12 days prior to ^89^Zr-oxine labeling. Cytotoxicity and cytokine release assays were performed 24 h following co-incubation of ^89^Zr-oxine labeled and unlabeled non-transduced (mock) T-cells and huLym-1-A-BB3z-CAR T-cells with target Raji cells. Cell viability counts were measured at 3, 24, 48, and 120 h, corresponding to day 0, 1, 2, and 5, following ^89^Zr-oxine labeling. In vitro outcome measures were performed in triplicate samples.

The study design for the in vivo experiment evaluating the biodistribution of varying doses of ^89^Zr-oxine labeled CAR T-cells is shown in Fig. 1b. Three groups were employed, wherein Raji-bearing NSG mice were randomized to receive a low (n = 3 mice), middle (n = 5 mice), or high dose (n = 3 mice) of ^89^Zr-oxine labeled huLym-1-A-BB3z-CAR T-cells. Actual doses administered into mice were as follows: Low dose (126.7 ± 2.5 kBq, 1.87 ± 0.04 × 10^6^ cells), Middle dose (542.6 ± 92.1 kBq, 7.14 ± 0.45 × 10^6^ cells), and High dose (1491.7 ± 36.1 kBq, 16.83 ± 0.41 × 10^6^ cells). Two experimental batches were performed to achieve the sample sizes for the three groups. For each experimental batch, 6 NSG mice were inoculated with Raji cells subcutaneously on the right flank 12 days prior to the administration of ^89^Zr-oxine labeled CAR T-cells. For all studies, mice received labeled CAR T-cells intravenously (IV) via the tail vein on day 0. Mice then underwent simultaneous PET/MRI imaging at 3, 24, 48, and 120 h post injection of ^89^Zr-oxine labeled cells, corresponding to day 0, 1, 2, and 5, respectively. Regions of interests (ROIs) were dissected on day 6 post-injection and gamma counted for ex vivo biodistribution quantitation. Percent injected dose per gram tissue (%ID/g) for ROIs, such as blood, bone, brain, liver, lungs, spleen, and tumor were derived from in vivo PET images and ex vivo biodistribution. We also calculated the in vivo tumor-to-blood ratio by dividing the tumor %ID/g by the blood %ID/g, to account for the accumulation of cell signal within the extravascular spaces of the tumor excluding signal from the blood vasculature compartment.

### Primary human T-cell isolation and CAR T-cell preparation

#### Primary human T-cell isolation

Human primary T-cells were isolated from peripheral blood mononuclear cells (PBMCs), obtained from human buffy coat preparations (Zenbio Inc.), using Ficoll-Paque (Life Technologies Inc., Carlsbad, CA) followed by T-cell isolation using the EasySep Human T-Cell Isolation Kit (Stem Cell Technologies, Seattle, WA). Isolated cells were then cultured in T-cell medium (43% Clicks, 43% RPMI 1640, 2% Glutamax (Life Technologies, Inc., Carlsbad, CA), 10% dFCS (Hyclone Laboratories), 1% non-essential amino acid solution, 1% pen/strep solution, 50 ng/mL IL-7-Fc, 50 ng/mL IL-15-Fc. Both the IL-7 and IL-15 reagents were produced in the Epstein Laboratory and enriched T-cells were frozen for subsequent use in liquid nitrogen.

#### CAR T-cell preparation

**Figure 2:**
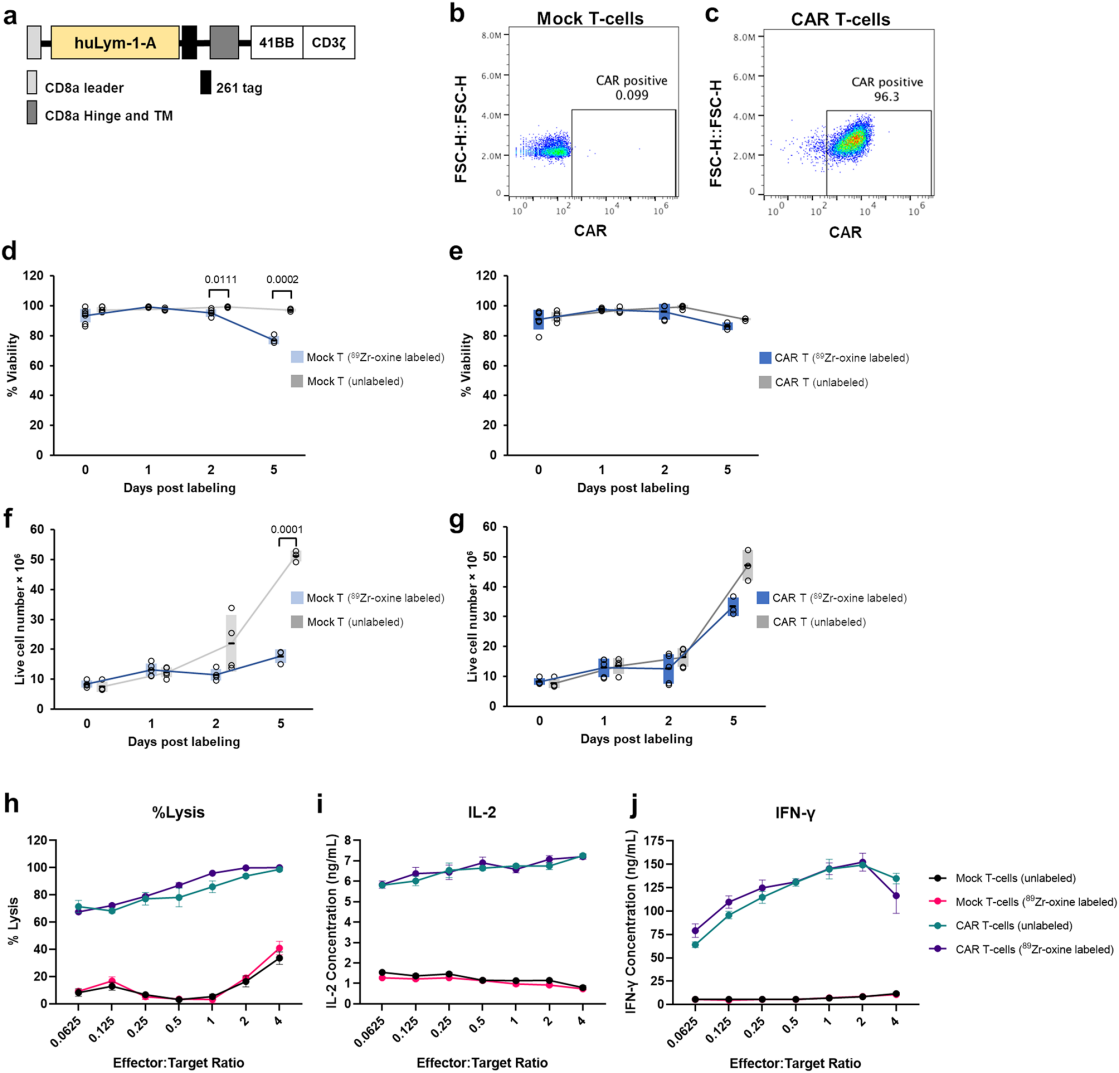
^89^Zr-oxine labeling does not significantly alter cell health and function of huLym-1-A-BB3z-CAR T-cells. (**a**) Schematic of 2nd generation CAR construct used for huLym-1-A-BB3z-CAR T-cells. Adapted from Zheng, et al., 2020 with permission of the copyright owner. (**b**, **c**) Flow cytometry analysis of CAR expression on non-transduced (mock) or transduced primary human T-cells (huLym-1-A-BB3z-CAR T-cells) on day 7. CAR expression was measured using Dylight 650-conjugated antibody against the 261 tag. (**d**, **e**) Percent viability measurement of subcultured ^89^Zr-oxine labeled and unlabeled mock and huLym-1-A-BB3z-CAR T-cells on day 0, 1, 2, and 4 time points, which correspond to 3, 24, 48, 120 h after ^89^Zr-oxine labeling, respectively. (**f**, **g**) Total live cell count (× 10^6^) of subcultured ^89^Zr-oxine labeled and unlabeled mock and CAR T-cells on the same time points. Viability and live cell number plots show dash lines as means, colored bars as standard deviation ranges, and open circles as individual data points. Significant differences are shown with p-values above brackets. A value of *p* < 0.05 was used to indicate statistical significance. (**h**) Cytotoxicity of ^89^Zr-oxine labeled and unlabeled mock and huLym-1-A-BB3z-CAR T-cells against Lym-1-positive Raji cells. (**i**, **j**) IL-2 and IFN-γ cytokines released by ^89^Zr-oxine labeled and unlabeled mock and huLym-1-A-BB3z-CAR T-cells. Cytotoxicity and cytokine release plots show means (closed circles) and standard deviation error bars. Labeling with ^89^Zr-oxine show unaltered CAR T-cell cytotoxicity and IL-2 and IFN-γ cytokine release.

Second generation CAR T-cells were constructed following procedures in Zheng et al*.*^21,22^ by transducing human primary T-cells with huLym-1-A-BB3z-CAR encoding lentivirus. The CAR sequences consisted of a binding domain (anti huLym-1-A ScFv), hinge and transmembrane domains from human CD8a, and a signaling domain from 4-1BB and CD3ζ (BB3z) (Fig. 2a). A ten amino acid epitope “AVPPQQWALS” (261-tag), derived from human placenta growth factor was inserted between the ScFv and CD8a hinge to enable CAR detection using an in-house antibody (anti-261-tag).

Twelve days prior to scheduled ^89^Zr-oxine labeling, primary human T-cells were thawed, enriched for three days, and were activated by adding CD3/CD28 beads (Life Technologies, Inc., Carlsbad, CA) at a 1:1 ratio. Primary human T-cells were then transduced by centrifugation with lentivirus (800 g, 90 min, MOI = 15) and LentiBlast (OZ Bioscience, San Diego, CA) followed by 24 h media change. Cells were then transferred to 24-well G-Rex plates (Wilson Wolf, St. Paul, MN) supplemented with fresh T-cell medium. CAR virus transduction efficiency was evaluated by flow cytometry (Applied Bio Systems Attune acoustic focusing cytometer) at day 7 using Dylight-488 conjugated anti-261-tag antibody. Transduced CAR T-cells were then enriched by magnetic sorting and reactivated with CD3/CD28 beads for further expansion. Cells from 5–6 days after reactivation were used.

#### Magnetic sorting

Up to 20 million CAR transduced cells were washed with PBS containing 0.5% FBS and 2 mM EDTA and stained with anti-261-tag antibodies at 4 °C for 15 min. Cells were washed and then 20 μl anti-Mouse IgG microbeads (Miltenyi Biotec, USA) were added. Bead-labeled cells were then enriched using a LS column (Miltenyi Biotec, USA).

### ^89^Zr-oxine cell labeling

#### ^89^Zr-oxine preparation

The ^89^Zr-oxine cell labeling procedure was adapted from Weist et al*.*^7^). ^89^Zr arrived in 1 M oxalic acid (3D Imaging, Inc., Arkansas, USA). One mg/mL oxine-HEPES solution was prepared using 10 mg 8-hydroxyquinoline (oxine, Sigma Aldrich, USA) in 0.1 mM HEPES in a glass scintillation vial with a PTFE top. The scintillation vial was placed and shaken in heated water for 5 min for oxine to fully dissolve, then allowed to passively cool to room temperature. For every 74 kBq (20 μCi) of ^89^Zr in oxalic acid, 1 ug of oxine was used to generate an ^89^Zr-oxine solution. To yield the ^89^Zr-oxine solution, 50 uL oxine-HEPES solution followed by 37 MBq (1 mCi) of ^89^Zr in oxalic acid were pipetted into a 0.3 mL glass LVI vial (Wheaton 225220-01) before being neutralized to pH 7.4 (measured using a pH indicator test (MColorpHast, Millipore, USA)) with 200 uL 1 M HEPES. Instant thin layer chromatography (iTLC) was then used to validate ^89^Zr-oxine conjugation. Two uL ^89^Zr-oxine solution or oxine-HEPES solution was aliquoted onto iTLC strips (Sigma Aldrich, USA) which were then placed in a 50 mL centrifuge tube with 5 mL of diluent in the dark for 15 min (PBS or EBWC solution – ethyl acetate (80% v/v), butanol (10% v/v), distilled water (5% v/v), and citrate (5% v/v)). Ultraviolet light exposed on the iTLC strip showed oxine-HEPES solution migrated along the solvent front, whereas ^89^Zr-oxine remained at the origin. This was validated by observing that the radioactivity remained near the origin on the iTLC strip as measured using a Ludlum Geiger counter (counts per minute) and a custom lead brick configuration.

#### ^89^Zr-oxine cell labelling

To achieve radiolabeled cells with specific activities (CSA) of 37–74 kBq (1–2 μCi) per million cells, 2.3 times the needed ^89^Zr-oxine activity was added to prepared cells (20 × 10^6^ cells/mL) in a 15 mL tube in Hanks’ buffered saline solution (HBSS) (assuming 50% activity retention in cells and 30% activity loss from transferring using plastic pipette tips). After gentle mixing and simple incubation for 30 min at 37 °C, cell culture medium was used to spin and wash the ^89^Zr-oxine labeled cells three times (5 min, 1227 × g, LW Scientific). Cells for IV injection in mice were then resuspended in HBSS and prepared in 30G insulin syringes before IV injection in mice. Aliquots of ^89^Zr labeled and unlabeled cells were resuspended in cell media in 25 mm^2^ cell culture flasks (EasYFlask, Thermo Scientific) and kept in 37 °C for subsequent in vitro viability and live cell count measurements, which paralleled the in vivo PET/MRI time points.

#### Activity retention and cell specific activity

Percent retention of ^89^Zr-oxine label in cells was determined by dividing the radioactivity in cell pellets after washing by the initial radioactivity added to the cell suspension and multiplied by 100. Radioactivity was measured using a CRC-25R dose calibrator (Capintec, Inc., adjusted for ^89^Zr^23^). Final CSA was measured by dividing the final activity retained in the cell pellet by the live cell count number obtained immediately after labeling.

### Evaluation of in vitro cell health and function

#### Viability and live cell count

Unlabeled and ^89^Zr-oxine labeled cells were subcultured and the total live cell number and %viability were measured (Moxiflo, Orflo) before and at 3, 24, 28, and 120 h after labeling. The viability and cell count assay used a cell membrane exclusion method with propidium iodide incubation of cells per manufacturer’s protocol.

#### Cytotoxicity assays

A luminescent-based cytotoxicity assay was performed following ^89^Zr labeling of mock and CAR T-cells. Raji-eGFP/Luc cells were used as target cells and ^89^Zr labeled and unlabeled mock and CAR T-cells were used as effector cells. Luminescent read-outs from Raji cells without effector cells were used as a control. Two-fold serial dilutions of 0.2 million Raji target cells were used to generate a standard curve to correlate live cells to luminescent signal output. Live target cell number after 24 h incubation at 37 °C with effector cells was calculated by correlating the luminescent signal to the standard curve. Wells were incubated with luciferase at room temperature in the dark for 10 min and immediately scanned using BIOTEK Synergy HT plate reader at 450 nm. The percentage of cell lysis was calculated by the following formula: % Lysis = [(# Raji cells in control wells − # Raji cells in effector wells) / (# Raji cells in control)] × 100%.

#### Cytokine secretion assay

Following 24 h incubation of Raji target cells with ^89^Zr-oxine labeled and unlabeled effectors cells, supernatant samples were collected for a cytokine secretion assay using enzyme-linked immunosorbent assay (ELISA) measurement per manufacturer’s instructions. One hundred µL of IL-2 and interferon gamma (IFN-γ) antibodies (eBioscience, Inc.) at 2 µg/mL were used to coat each well on the 96-well plates. The plate was wrapped in parafilm and kept in a 4 °C refrigerator overnight. The fluid was then discarded and 200 µL of blocking buffer (1% BSA in PBS) was added to each well and incubated for 2 h at room temperature. Wells were then washed with PBS 3 times. Supernatant samples at 100 µL volumes were added and incubated for 1 h at room temperature followed by 3 washes with PBS. Biotinylated secondary antibodies for IL-2 and IFN-γ (eBiosciences, Inc.), respectively, were added at 500 µg/mL and incubated for 1 h. After 3 washes with PBS, wells were incubated with a streptavidin tertiary antibody (500 µg/mL) for 1 h and washed again with PBS 3 times. Wells were then incubated with chemiluminescent substrate solution for 30 min in the dark and plates were read at 450 nm using a BIOTEK Synergy HT.

### MRI and PET image acquisition

#### Image acquisition

MRI and PET images were acquired using a 7 Tesla MRI scanner with an integrated PET camera (MR solutions, Guildford, UK). Animals were anesthetized with isoflurane (1.5–2%) and respiration was monitored using a pneumatic pillow. Body temperature was maintained at 37 °C using a heated animal holder (Minerve, France). Two-dimensional Fast Spin Echo (FSE) transverse T1-weighted MR images were acquired to identify define anatomy with the following parameters: TE/TR = 11 ms/3556 ms, slice thickness = 1 mm, 1 average, echo train length = 4, field of view = 72 mm × 72 mm, matrix size = 512 × 256, flip angle = 90°. For PET, a list mode static acquisition protocol was obtained for 10 min at the 3, 24, and 48 h time points, and 20 min at 120 h. Image data were corrected for intrascan radiodecay, detector nonuniformity, and random coincident noise. 3D-OSEM reconstruction was performed, resulting in a 0.28 × 0.28 × 0.28 mm^3^ voxel dimension, 34 mm field of view, 64 subsets, and 1 iteration.

#### Image analysis

**Figure 3:**
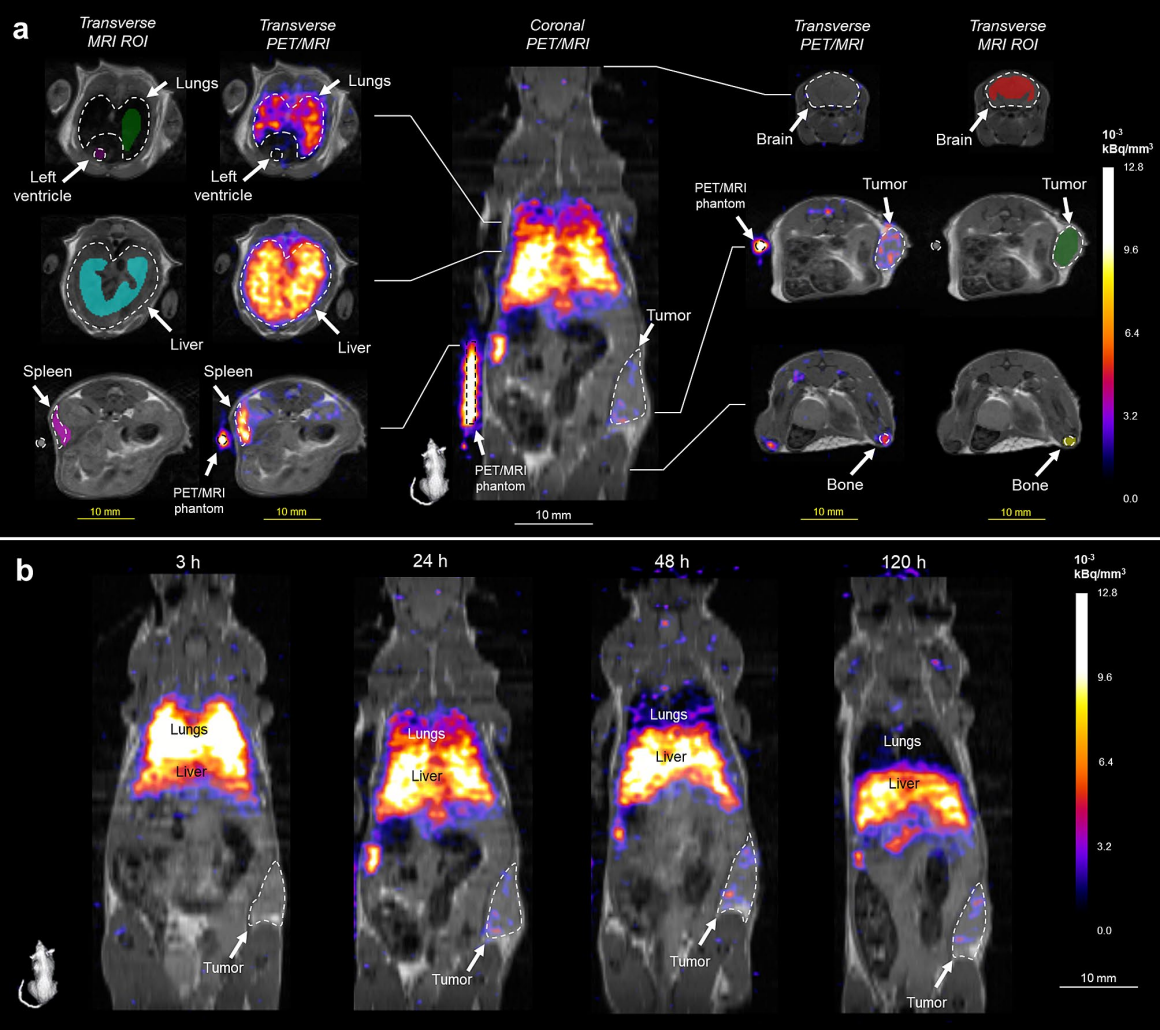
Representative PET/MRI of ^89^Zr-oxine labeled huym-1-A-BB3z-CAR T-cells. (**a**) Central figure shows a representative coronal PET (colored: nih fire) and MRI (gray) overlay image of a Raji-bearing NSG mouse 24 h after IV administration of ^89^Zr-oxine labeled huLym-1-A-BB3z-CAR T-cells (1517 kBq, 16.4 × 10^6^ cells). Mouse image on the lower left shows orientation of coronal slice. Medial columns of transverse cross-sections show PET/MRI overlay images with slice positions indicated along the central coronal PET/MRI image. Lateral columns of transverse cross-sections show the same MRI slices with manually defined regions of interests (ROI) in color within the delineated organ or tissue boundaries in dashed lines. ROIs analyzed were the lungs (green), left ventricle for blood (dark magenta), liver (cyan), spleen (magenta), brain (red), tumor (dark green), and bone (yellow). ROI activity concentrations were obtained from PET images and expressed as percent injected dose per gram of tissue for CAR T-cell biodistribution analyses. Scale bars indicate 10 mm for both coronal (white) and transverse images (yellow). Color bar indicates PET activity concentration in × 10^–3^ kBq/mm^3^ units. (**b**) Representative coronal PET/MRI slices at the 3, 24, 48, and 120 h timepoints following IV injection of huLym-1-A-BB3z CAR T-cells in same mouse from (**a**). Mouse on the lower left shows orientation of coronal images. Activity was observed highest in the lungs at 3 h and diminished overtime, while activities in liver and spleen increased at the later time points. Activity accumulation in tumor was evident by 24 h following ^89^Zr-oxine labeled CAR T-cell injection. Color bar indicates PET activity concentration in × 10^–3^ kBq/mm^3^ units. White scale bar on lower right indicates 10 mm.

Regions of interest (ROI) activities in kBq/mm^3^ were obtained using overlaid ROIs from MRI onto PET images using VivoQuant 4.0 software for Windows 64-bit, (Invicro, Inc., Boston, MA, USA, www.vivoquant.com). PET and MRI images were validated using the PET/MRI phantom, which consisted of a 1 cm PE-60 tube filled with 10 µL of ~ 18.5 kBq ^89^Zr-oxine solution. ROIs included in the analysis are blood, bone, brain, liver, lungs, spleen, and tumor. The left ventricle space was identified and denoted as the blood ROI. Representative drawing of the ROIs and the delineation of the ROI boundaries in a Raji-bearing NSG mouse are shown in Fig. 3a at the 24 h timepoint following IV injection of ^89^Zr-oxine labeled huLym-1-A-BB3z CAR T-cells. Figure 3b shows representative coronal PET/MRI slices at the 3, 24, 48, and 120 h timepoints following IV injection of huLym-1-A-BB3z CAR T-cells in same mouse from Fig. 3a.

### Ex vivo biodistribution

On day 6 post injection of ^89^Zr-oxine labeled CAR T-cells, mice were euthanized by isoflurane overdose and cervical dislocation. Mice were generously sprayed with 70% ethanol solution for disinfection and placed on a dissecting tray with pins. The abdominal wall was opened below the rib cage using surgical scissors. The sternum was lifted with forceps and diaphragm was cut, and then the lower part of the ribcage was cut to partially expose the heart. Blood was drawn by inserting the needle of a 1 mL syringe, with 23G needle prepared with 15 μL 0.1 M EDTA pH 7.4, to the right atrium and slowly pulling the plunger, adjusting the needle to be patent and unblocked by heart tissue. Blood was drawn over one minute and transferred to pre-weighed polypropylene tubes. Then organs and tissues of interests (femoral bone, brain, liver, lungs, spleen, and tumors) were dissected. All samples were transferred to pre-weighed polypropylene tubes and weighed again to obtain actual sample weights. Gamma counting were done at the USC Molecular Imaging Core on a PerkinElmer Wizard 2470 gamma counter. ^89^Zr-oxine standards were generated by serial dilution of ^89^Zr-oxine solution, measured with the CRC-25R dose calibrator (Capintec, Inc.) and were also gamma counted alongside experimental samples. Gamma counts were converted to %ID/g for analysis. All radioactive samples were decayed in lead storage prior to discarding in biohazard or regular waste receptacles.

### Statistical analysis

Data are presented as mean ± standard deviation (SD). Unpaired, two-tailed, t-tests with individual variances computed for each comparison were used between labeled and unlabeled mock and CAR T-cells in the in vitro cell health and function assays. The Holm-Sidak’s method was used to correct for multiple comparisons. A mixed-effect model, with sphericity assumed, was performed for in vivo biodistribution comparisons over time. Tukey corrected post hoc comparisons between doses for each time point were performed. One-sample t-test, one-tailed, with unequal variances against 0.6 threshold was performed for tumor-to-blood ratios to test for enhanced uptake of CAR T-cells in extravascular tumor tissue. For ex vivo biodistribution analysis, unpaired t-test, with individual variances computed for each dose comparison for each region was performed using the Holm-Sidak’s correction for multiple comparisons. A value of *p* < 0.05 was used to indicate statistical significance. Statistically significant findings are indicated in the figures with p-values shown above brackets. All statistical analyses were performed using GraphPad Prism version 8.4.3 for Windows 64-bit (GraphPad Software, San Diego, CA, USA, www.graphpad.com).

## Results

### In vitro cell health and function assays following ^89^Zr-oxine labeling

Figure 2b,c shows the flow cytometry analysis of CAR expression on mock and transduced primary human T-cells, respectively, demonstrating successful CAR transduction on the primary human T-cells. To determine that ^89^Zr-oxine labeling had no detrimental effect on cell health, we subcultured ^89^Zr-oxine labeled and unlabeled mock and huLym-1-A-BB3z-CAR T-cells for percent viability (%viability) and total live cell number measurements over the same time points as the in vivo imaging. ^89^Zr-oxine labeled mock T-cells showed significantly less %viability compared to unlabeled mock T-cells on day 2 (*p* = 0.0111) and 5 (*p* = 0.0002) post labeling (Fig. 2d), while %viability was not significantly different between ^89^Zr-oxine labeled and unlabeled CAR T-cells (Fig. 2e). We observed a significant reduction in the number of live cells on day 5 post labeling (*p* = 0.0001) in ^89^Zr-oxine labeled mock T-cells (Fig. 2f), However, no significant effect of ^89^Zr-oxine labeling was observed in the huLym1-A-BB3z-CAR T-cells in the total live cell counts (Fig. 2g). We also determined whether cell function was affected by ^89^Zr-oxine labeling. We observed no significant differences in cell function between ^89^Zr-oxine labeled and unlabeled mock and CAR T-cells after measuring the cytotoxicity (Fig. 2h) and the secreted IL-2 and IF-γ cytokines (Fig. 2i,j) following 24 h incubation of the effector T-cells with target Raji-GFP/Luc cells.

### In vivo PET imaging of escalating doses of huLym-1-A-BB3z-CAR T-cells

**Figure 4:**
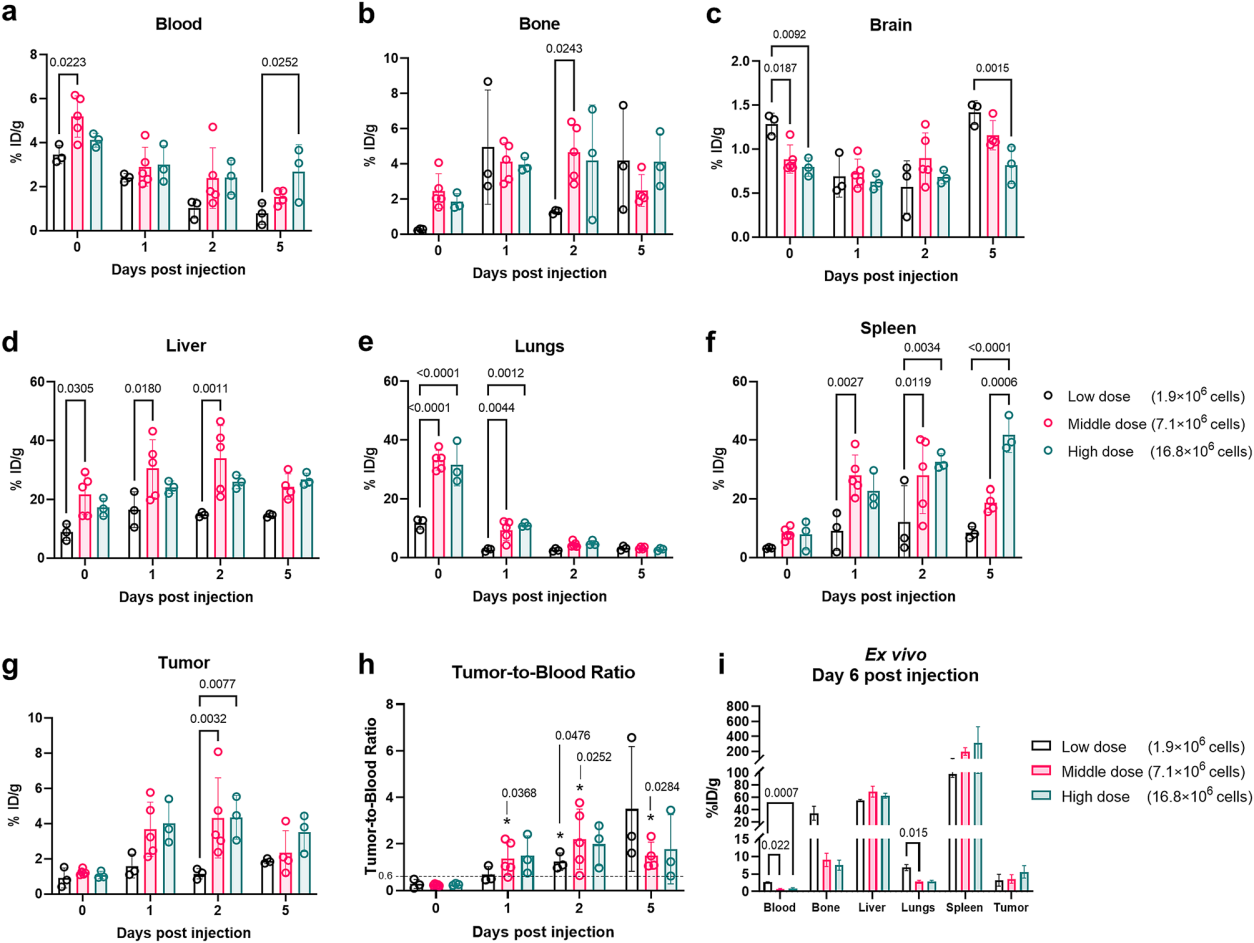
Tissue biodistribution of ^89^Zr-oxine labeled huLym-1-A-BB3z-CAR T-cells dose escalation. Significant changes in %ID/g overtime were observed in all regions. (**a**–**g**) In vivo blood, bone, brain, liver, lungs, spleen, and tumor %ID/g of Raji-bearing NSG mice administered with low (mean ± SD: 126.7 ± 2.5 kBq, 1.87 ± 0.04 × 10^6^ cells), middle (542,6 ± 92.1 kBq, 7.14 ± 0.45 × 10^6^ cells) or high dose (1491.7 ± 36.1 kBq, 16.83 ± 0.41 × 10^6^ cells) of ^89^Zr labeled huLym-1-A-BB3z-CAR T-cells (Low dose, n = 3 mice; Middle dose, n = 5 mice; High dose, n = 3 mice). Columns with error bars indicate means and standard deviations (SD) and open circles as individual data points. (**h**) Tumor-to-blood ratio obtained by dividing tumor %ID/g by blood %ID/g to account for cell signal accumulation in extravascular tumor tissue. Significant cell accumulation was observed by beginning on day 1 to day 5 in NSG mice that received the middle dose, and in mice that received low dose on day 2, by one-sample t-test vs. 0.6 threshold to overcome the enhanced permeability and retention (EPR) effect. (**i**) Ex vivo biodistribution %ID/g of low, middle, and high CAR T-cell doses on day 6 post CAR T-cell injection. Columns and error bars indicate means and SD, respectively. Statistically significant findings between comparisons are indicated with p-values above brackets. A value of *p* < 0.05 was used to indicate statistical significance.

We did not observe toxicity reactions in the mice acutely and over the time points of the in vivo experiment, up to 7 days after administration of the ^89^Zr-oxine labeled huLym1-A-BB3z-CAR T-cells. We performed a mixed-effect analysis of the tissue accumulation of ^89^Zr-oxine labeled CAR T-cells by ROI using time (day post-injection) and dose level (low, middle, or high) as fixed effects. Activity %ID/g by ROI overtime is shown in Fig. 4. We observed changing activity %ID/g levels overtime in all regions, which were reflected in the observed significant main effect of time in the mixed-effect model analysis (blood: [F(3, 23) = 23.30, *p* < 0.0001], bone: [F(3, 23) = 5.994, *p* = 0.0036], brain [F(3,23) = 14.97, p < 0.0001], liver: [F(3, 23) = 11.75, *p* < 0.0001], lungs: [F(3, 31) = 164.5, *p* < 0.0001], spleen: [F(3, 23) = 18.70, *p* < 0.0001], and tumor: [F(3, 23) = 16.92, *p* < 0.0001]). We then examined the dose level effects by ROI. In blood (Fig. 4a), a main effect of dose level was observed [F(2,8) = 4.714, *p* = 00444]. Significantly lower blood %ID/g was detected on day 0 in the Low dose group compared to the Middle dose group (p = 0.0233) and significantly less blood %ID/g compared to the High dose group (*p* = 0.0252) on day 5 post ^89^Zr-oxine labeled CAR T-cell injection. In bone (Fig. 4b), while we did not observe an overall effect of dose effect levels, we observed bone %ID/g in Middle dose group higher compared to Low dose group on day 2 (*p* = 0.0243). In brain (Fig. 4c), there was a time × dose interaction [F(6,23) = 4.097, *p* = 0.0061], where Low dose brain %ID/g showed significantly higher levels compared to the Middle (*p* = 0.0187) and High dose (*p* = 0.0092) groups on day 0 and significantly more compared to the High dose group on day 5 (*p* = 0.0015). In liver (Fig. 4d), we observed a dose main effect [F(2,8) = 5.233, *p* = 0.0352], with %ID/g in the Middle dose group means significantly higher than the Low dose group on day 0 (*p* = 0.0305), day 1 (*p* = 0.0180), and day 2 (*p* = 0.0011) post injection. In lungs (Fig. 4e), a significant effect of dose level [F(2,31) = 35.75, *p* < 0.0001] and time × dose interaction [F(6,31) = 14.28, *p* < 0.0001] were observed. Low dose group initially demonstrated significantly lower lung %ID/g on day 0 (3 h post injection) compared to the Middle (*p* =  < 0.0001) and High dose (*p* < 0.0001) groups, as well as on day 1 post injection when compared to the Middle (*p* = 0.0044) and High dose (*p* = 0.0012) groups. By day 2 and day 5 post injection activity in lungs decreased, with %ID/g levels comparable between all groups. In spleen (Fig. 4f), there were significant main effect of dose level [F(2,8) = 11.91, *p* = 0.0040] and a time × dose interaction [F(6,23) = 4.782, *p* = 0.0027]. We observed increasing accumulation overtime of ^89^Zr-oxine labeled CAR T-cells, with significantly higher %ID/g in Middle dose group compared to Low dose group on day 1 (*p* = 0.0027) and day 2 (*p* = 0.0119) post injection, and significantly higher %ID/g in the High dose group compared to the Low dose group on day 2 (0.0034) and day 5 (*p* < 0.0001). We detected higher spleen %ID/g in the High dose group versus Middle dose only on day 5 (*p* = 0.0006) post injection. Lastly, in tumor (Fig. 4g), although no main effect by dose level was observed, there was a significant time × dose level interaction [F(6,23) = 2.907, *p* = 0.0294]. Multiple post hoc comparisons showed significantly higher tumor %ID/g accumulation in the Middle dose group (*p* = 0.0030) and in the High dose group (*p* = 0.0075) than the Low dose group on day 2 post injection. Tumor-to-blood ratio (Fig. 4h) showed significant cell accumulation in the extravascular tumor region (> 0.6), by one-sample t-test, in NSG mice that received the low dose 2 days post ^89^Zr-oxine labeled CAR T-cell injection (*p* = 0.0476). Significant tumor accumulation was detected in mice that received the middle dose beginning on day 1 (*p* = 0.0368) that is observed on all subsequent time points, on day 2 (*p* = 0.0252) and day 5 (*p* = 0.0504), following ^89^Zr-oxine labeled CAR T-cell injection. While mean tumor-to-blood ratios observed in the High dose group were greater than the 0.6 threshold, no statistically significant cell accumulation was observed in the High dose group.

### Activity retention and cell specific activity

**Figure 5:**
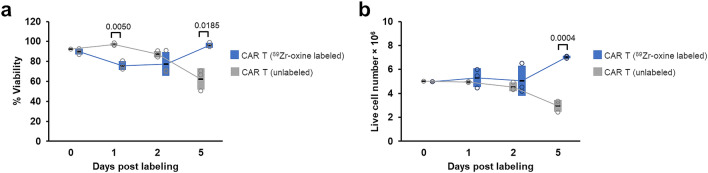
Parallel in vitro viability and live cell count of subcultured ^89^Zr-oxine labeled huLym-1-A-BB3z-CAR T-cells administered in mice. (**a**) Percent viability and **(b)** total live cell count (× 10^6^) of subcultured ^89^Zr-oxine labeled and unlabeled CAR T-cells from the same cell batch administered to live mice. Viability and live cell count plots show dash lines as means, colored bars as SD, and open circles as individual data points. Statistically significant findings between comparisons are indicated with *p* values above brackets. A value of *p* < 0.05 was used to indicate statistical significance.

Similar to previous studies^11^, we observed that the mean ± SD radiolabel retention and cell specific activity for the huLym-1-A-BB3z-CAR T-cells was 33.5 ± 3.8% and 65.7 ± 23.8 kBq/1 × 10^6^ cells, respectively, when taking the in vitro and in vivo experiments together. In the in vitro experiment, ^89^Zr-oxine labeling of CAR T-cells yielded 30.0% activity retention and a cell specific activity of 41.0 kBq/1 × 10^6^ cells. In the in vivo experiment, batch 1 of the ^89^Zr-oxine cell labeling resulted in 35.3% activity retention and CSA of 67.6 kBq/1 × 10^6^cells CSA, and batch 2 yielded 43.2% activity retention and 88.6 kBq/1 × 10^6^cells CSA. We observed that the actual initial amount of ^89^Zr-oxine added to huLym-1-A-BB3z-CAR T-cells used in the in vivo experiment were higher compared to ^89^Zr-oxine amount added to cells in the in vitro trial, which resulted in the higher activity retention and CSA of the ^89^Zr-oxine labeled CAR T-cells. The %viability and live cell number counts for the subcultured ^89^Zr-oxine labeled CAR T-cells for the two batches of in vivo trials were combined. Table 1 shows the number of cells at the start and end of each labeling batch for the in vivo and in vitro experiments, the calculated and actual initial and end activities used for labeling, activity retention and cell specific activities (CSA). The calculated initial activity was determined from preliminary labeling trials to achieve desired cell specific activity (CSA) of 37-74 kBq/1 × 10^6^ cells. We determined it was necessary to use 2.3 times more ^89^Zr-oxine activity to account for low retention and radiolabel loss during the labeling process. However, in the in vivo experiment, actual activities added to cells were higher than the calculated activities, which then contributed to higher CSA values. The significant decrease in %viability of ^89^Zr-oxine labeled huLym-1-A-BB3z-CAR T-cells (Fig. 5a) on day 1 (*p* = 0.0050) post labeling may be attributed to this higher starting amount of ^89^Zr-oxine and CSA. In contrast to the in vitro trial, we also observed higher %viability (*p* = 0.0185) (Fig. 5a) and live cell number (*p* = 0.0004) (Fig. 5b) in ^89^Zr-oxine labeled CAR T-cells compared to unlabeled CAR T-cells on day 5 post-labeling in the in vivo experiment.

**Table 1.** ^89^Zr-oxine labeling activity retentions and cell specific activities (CSA).

	In vitro	In vivo	In vivo
		Batch 1	Batch 2
Pre labeling cell number (× 10^6^)	40.0	45.0	90.0
Post labeling cell number^a^ (× 10^6^)	28.0	30.0	65.0
Calculated initial activity (kBq)	5106.0	5744.3	11,488.5
Measured beginning activity (kBq)	3829.5	6179.0	15,318.0
Measured ending activity^a^ (kBq)	1148.9	2027.7	5759.6
Activity retention (%)	30.0	32.8	37.6
CSA (kBq/1 × 10^6^ cells)	41.0	67.6	88.6

^a^1 h after measured beginning activity.

## Discussion

In this study, not only we demonstrated successful non-invasive assessment and monitoring of adoptively transferred immune cells in a preclinical model of human cancer using ^89^Zr-oxine cell imaging, but we also addressed whether there is a dose-dependency of the in vivo CAR T-cell targeting and biodistribution. Correlating dose to accumulation can provide efficacy information that could guide development of immunotherapy in the clinic. This method shows promise to provide real-time in vivo information regarding tumor uptake of CAR cells, cell persistence, and non-specific organ accumulation that can be correlated with treatment efficacy and negative side-effects.

We demonstrated successful labeling of huLym-1-A-BB3z-CAR T-cells with ^89^Zr-oxine that required no further purification. ^89^Zr-oxine cell retention in huLym-1-A-BB3z-CAR T-cells in this study was comparable to previous ^89^Zr-oxine cell labeling methods that involved extraction with chloroform^9,17,18^ or conversion to ZrCl_4_^11,13^. We also observed ^89^Zr-oxine cell retention of 33.5% in huLym-1-A-BB3z-CAR T-cells, whereas IL13Rα2-CAR T-cells retained 75% of the ^89^Zr-oxine label observed by Weist et al.^7^ using the same labeling method. This suggests that in addition to cell type, different CAR constructs can also contribute to differences in label retention. In vitro cell health measurements showed that ^89^Zr-oxine labeling did not significantly alter %viability and proliferation (live cell count) of CAR T-cells over 5 days following labeling. Mock T-cells, however, showed decreased %viability on day 2 and day 5 after labeling, and impaired increase in live cell count on day 5 post labeling. Cell function assays demonstrated that ^89^Zr-oxine labeling did not significantly alter tumor cell killing capacity and cytokine production of either mock or CAR T-cells. The delayed proliferation or increase in the live cell number of labeled and unlabeled CAR T-cells observed only on day 5 timepoint was expected because Zheng et al*.* 2020 already demonstrated that the BB3z signaling domain contributed to limited proliferation of CAR T-cells with humanized Lym-1, and replacing BB3z with DAP12 restored expansion capacity in the transduced T-cells^22^. Interestingly, the ^89^Zr-oxine labeled CAR T-cells used in the in vivo experiments had higher cell specific activities and showed a transient decrease in %viability on day 1 and an eventual enhanced %viability and live cell count on day 5 compared to unlabeled CAR T-cells. We hypothesize that the CAR construct used in this study, huLym-1-A-BB3z, may have interactions with other surface ligands that affect the CAR T-cells to be more resilient under radiolabeling conditions. We are the first to report the induced robustness in cell viability from radiolabeling of CAR transduced T-cells. For future studies that correlate CAR T-cell biodistribution to therapeutic efficacy, both cell health and functional measurements following ^89^Zr-oxine labeling, with CSA ≤ 41 kBq/1 × 10^6^ cells, should be performed in the same batch.

We also showed successful but low tumor accumulation of CAR T-cells. Middle and high dose CAR T-cells and CAR NK-92MI cells reached 1–5%ID/g in tumors in the in vivo PET imaging and ex vivo biodistribution (Fig. 4g,i), levels according to previous reports showed enhanced targeting of CAR cells versus mock cells^7^. We did not observe tumor regression, perhaps due to insufficient time (< 6 days) to observe therapeutic effects on tumor burden and the low accumulation of CAR T-cells in tumor significantly delayed by transit in first lung (3–5 h) and then liver and spleen (1–3 days). However, Zheng et al*.*^21,22^ reported significant regression of intraperitoneally (IP) implanted Raji tumors in NSG when Lym-1-directed CAR T-cells were IP administered. This reveals that the impairment of CAR infiltration into subcutaneous tumor implants, similar to solid tumors, may be due to limited tumor antigen presentation to CAR T-cells confounded by long transit times before reaching tumor, which can inactivate the cytotoxic function of injected cells^24,25^.

A limitation of ^89^Zr-oxine cell tracking is that the radiolabel may be liberated in vivo through apoptosis or cell expansion. It is possible the ^89^Zr-oxine label can be taken up by other phagocytotic cells, like macrophages, however, their biodistribution needs further investigation and is outside the scope of this study. Free ^89^Zr has been shown to bind plasma proteins, such as albumin in blood^26,27^. ^89^Zr bound to albumin can then accumulate in tumors via enhanced permeability and retention (EPR) effect and confound the tumor uptake signal from ^89^Z-oxine labeled cells^27^. ^89^Zr-oxine also has strong affinity for bones and joints, similar to ^89^Zr-oxalate and characteristic of free ^89^Zr itself^28^. Zr^4+^ has high affinity to phosphates, forming an insoluble salt with phosphate and precipitating in water. It is, therefore, likely that the phosphates in non-soft tissue, mineralized constituents in bone may be chelating free ^89^ZrL_4_ label^28^ and account for the bone-seeking nature of ^89^Zr.

To account for free ^89^Zr label, it is recommended to compare biodistribution of ^89^Zr-oxine labeled cells with the biodistribution of the ^89^Zr-oxine alone^27^. ^89^Zr-oxine label biodistribution, however, was not obtained in this study. Thus, we also presented the tumor-to-blood ratios to reflect non-vascular, specific tumor uptake of ^89^Zr-oxine labeled CAR cells, where tumor-to-blood ratio > 0.6 indicates positive cell accumulation. This baseline was inferred from Heneweer and colleagues’ measurement of the extent of albumin uptake in tumor xenographs via EPR, by measuring tumor-to-blood ratios of systemically administered ^89^Zr-albumin^27^. . NSG mice that received the low dose showed enhanced tumor uptake by day 2, while NSG mice administered with the middle dose of ^89^Zr-oxine labeled CAR T-cells showed significant tumor accumulation (tumor-to-blood ratio > 0.6) starting on day 1 up to day 5 post injection (Fig. 4h). While no significant cell accumulation was observed in the high dose group, which may be attributed to the small group size, tumor-to-blood ratios were greater than the 0.6 EPR effect threshold. Alternatively, systemic infusion of deferoxamine (Desferal), a stable hexadentate ^89^Zr chelator and used clinically for iron overload^29^, immediately following systemic injection of ^89^Zr-oxine labeled NK cells has been shown to quickly clear free ^89^Zr signal through kidneys in rhesus monkeys^14^. Further investigation is therefore necessary to determine the risk–benefit ratios associated with deferoxamine dosing with CAR cell therapy.

For activation and persistence, CAR T-cells require rapid exposure to their intended target and become functionally inactive within a short period of time if they do not encounter antigen positive targets. Therefore, it is important to understand the movement kinetics of CAR T-cells within the 3-dimensional space of the host. Success or failure of CAR T-cell immunotherapy depends on their transit through lung, liver, and spleen. This paper is the first of such work to describe the long transit time of CAR T-cells after administration in the lungs, then liver and spleen. Data shown here indicate that dosing may be an important consideration for optimizing the transit time of systemically administered CAR T-cells to tumor. Furthermore, the extent of early tumor uptake promises to be a useful outcome measure in future preclinical studies to assess the synergistic effects of treatments used in combination with CAR therapy on tumor regression and survival. Multimodal immunotherapy with CARs can include antibody-based treatments that can enhance CAR cell tumor infiltration^30,31^, activate endogenous anti-tumor responses and block T-cell inhibitory signals in the tumor microenvironment^32^.

The in vivo biodistribution and trafficking studies using PET/MRI imaging methods provided an early assessment of the targeting specificity of CARs and a method to evaluate differences in the biodistribution and trafficking of different CAR cell candidates for adoptive cell therapy. These CARs, when directly radiolabeled with ^89^Zr-oxine, are stable and remain viable, and offer a unique advantage as a useful preclinical tool for studying their uptake, biodistribution, and trafficking because they demonstrate sustained retention of radioactivity inside tumor target for at least 5 days. Furthermore, these efforts demonstrate that non-invasive PET imaging can provide real-time monitoring of the biodistribution of CAR T-cells in tumor tissues and most normal tissues. Therefore, radiolabeled CARs hold the promise of further extending their use in interpreting efficacy, off-target toxicities, and adverse effects in a particular tissue, in both the preclinical and clinical settings, and may identify the value of the site of administration and dosing schedules in optimizing cell therapies for the treatment of cancer and related diseases.

## Conclusions

^89^Zr-oxine can be used to track and monitor real-time in vivo biodistribution of T-cells non-invasively. This study provides proof of concept that molecular imaging with PET using ^89^Zr-oxine labeled CAR T-cells, which is a clinically applicable technique, can play an important role in early drug development. ^89^Zr-oxine cell imaging can provide decisive information about tumor-targeting kinetics, efficacy, and potential adverse effects of CAR T-cells.
